# Adoption and Barriers to CAD/CAM Technology Among Dentists in the Dominican Republic: A Cross-Sectional Study

**DOI:** 10.1016/j.identj.2026.109411

**Published:** 2026-04-02

**Authors:** Patricia Grau Grullón, Rodrigo Varella Carvalho, Gabriela Velázquez, Leyani Pérez, Roxana Crespo, Giana Lima

**Affiliations:** a Centro de Investigación en Biomateriales y Odontología, Universidad Iberoamericana (UNIBE), Santo Domingo, Dominican Republic; b Department of Dentistry, Institute of Life Sciences, Federal University of Juiz de Fora (UFJF), Governador Valadares, Minas Gerais, Brazil; c Faculty of Dentistry, Federal University of Pelotas (UFPel), Pelotas, Rio Grande do Sul, Brazil

**Keywords:** CAD-CAM, Dental technology, Questionnaires, Cross-sectional studies, Dental prosthesis design, Dentist

## Abstract

**Introduction:**

Computer-aided design and computer-aided manufacturing (CAD/CAM) have transformed modern dentistry, yet evidence from the Caribbean remains limited.

**Objective:**

To assess CAD/CAM adoption among dentists in the Dominican Republic and identify associated demographic and professional factors, barriers, and future adoption intentions.

**Methods:**

A nationwide cross-sectional online survey was administered to dentists registered with the Dominican Dental Association between June and November 2024. A validated questionnaire was distributed through professional networks and social media. Data were analysed using descriptive statistics, chi-square tests, and multivariable logistic regression to examine associations between adoption and specialty, practice type, postgraduate education, and years since graduation.

**Results:**

A total of 374 dentists responded; the adoption rate was 39.3%. Adoption was significantly associated with specialty and practice type (*P* < .001), but not with years since graduation (*P* = .740) or geographic region (*P* = .464). Prosthodontists (odds ratios [OR] = 9.16) and orthodontists (OR = 7.50) were the most likely adopters, while dentists in public institutions (OR = 0.18) and academia (OR = 0.28) were less likely to use CAD/CAM. Among nonusers, major barriers included high costs (41.9%). However, 71.4% expressed intention to adopt CAD/CAM.

**Conclusions:**

Adoption of CAD/CAM in the Dominican Republic is shaped by specialty and practice setting but limited by financial and educational barriers. Expanding training opportunities and reducing costs may facilitate more equitable digital integration.

**Clinical Significance:**

Understanding determinants of CAD/CAM adoption can guide targeted training and policy strategies to improve efficiency and care quality in resource-limited regions.

## Introduction

Digital technologies are reshaping contemporary dental practice, shifting workflows from analogue impressions and manual fabrication towards fully integrated digital systems. Intraoral scanners and computer-aided design and computer-aided manufacturing (CAD/CAM) platforms improve precision, efficiency, and treatment customization, while advances in ceramic and hybrid materials continue to expand clinical indications.^1^, ^2^, ^3^ Complementary tools such as three-dimensional printing further enhanced diagnostic and laboratory procedures, including the fabrication of surgical guides and models.^4^^,^^5^

Digital innovation has become a key driver in strengthening oral healthcare systems and expanding access to high-quality care worldwide, and recent analyses highlight the accelerating integration of digital clinical workflows into routine clinical practice.^6^, ^7^, ^8^

Despite these advances, CAD/CAM adoption remains uneven, influenced by practice setting, specialty, training exposure, and economic feasibility. Surveys from regions such as the United Kingdom, India, Saudi Arabia, China, Egypt, and Austria identify positive attitudes towards digital dentistry but note persistent obstacles, including high costs, limited training opportunities, and infrastructural constraints, particularly in smaller or public-sector clinics.^9^, ^10^, ^11^, ^12^, ^13^ Training gaps remain globally, with many clinicians relying on short courses or self-guided learning instead of structured instruction.^14^^,^^15^ Differences by specialty are frequently observed, with prosthodontists and orthodontists reporting the highest adoption rates.^13^ Younger dentists often demonstrate greater readiness to integrate digital workflows, although adoption does not always translate into implementation under cost-limited conditions.^14^^,^^16^

Although digital dentistry has expanded globally, the Caribbean remains notably absent from published research on CAD/CAM adoption. Socioeconomic disparities, variable access to technology, and structural differences between public and private healthcare systems may shape CAD/CAM integration. The Dominican Republic, with nearly 11,000 practising dentists and one of the largest dental education networks in the Caribbean, offers an important context to explore these issues.

Therefore, this study aimed to evaluate the adoption of CAD/CAM technology among dentists in the Dominican Republic, identify demographic and professional predictors of use, and explore perceived barriers and future adoption intentions. The null hypothesis stated that CAD/CAM adoption would not be influenced by these characteristics.

## Materials and methods

### Study design and ethical approval

This cross-sectional observational study surveyed dentists practising in the Dominican Republic between June and November 2024, adhering to the SUrvey Reporting GuidelinE (SURGE).^17^ The protocol was approved by the Research Ethics Committee of Universidad Iberoamericana (approval code: CEI2024-0589). This study was conducted in accordance with the Declaration of Helsinki. Participation was voluntary and anonymous, and digital informed consent was obtained from all respondents prior to survey completion.

### Study population and sampling strategy

The target population consisted of all dentists registered with the Dominican Dental Association (*n* = 11,108). A minimum sample size of 372 was calculated using OpenEpi (Version 3.01), based on a 95% confidence level, 5% margin of error, and an expected response distribution of 50%.

Eligible participants were invited to complete a self-administered online questionnaire via Google Forms. The survey link was distributed through professional WhatsApp groups, Instagram, and institutional email lists. Because these channels were open-access, the exact number of individuals who received the invitation could not be determined. Using the total number of registered dentists as the maximum possible sampling frame, the minimum estimated response rate was 3.3% (372/11,108). As with most web-based surveys, the study is subject to self-selection and recruitment bias.

National statistics describing the distribution of dentists by region, practice type, or years since graduation are not available; therefore, a formal representativeness analysis could not be performed. However, the sample includes respondents from all major regions of the country, providing broad geographic national coverage. Dentists practising abroad were excluded; no additional exclusion criteria were applied.

### Survey instrument and validation

The questionnaire was adapted from a previously published instrument developed by Krastev et al,^16^ with permission obtained via email from the original authors. It was translated into Spanish by two independent bilingual professionals with dental backgrounds: one native English speaker fluent in Spanish and the other a native Spanish speaker fluent in English. A back translation was performed, followed by a blind review conducted by two CAD/CAM experts fluent in English. Semantic and conceptual equivalence was confirmed, and minor adjustments were made by consensus.

Content validation was performed through expert judgment by five dental professionals experienced in CAD/CAM technology. Each item was rated for sufficiency, clarity, and relevance using a 4-point Likert scale. All items achieved average scores above 3.5 in each category, confirming adequate content validity. The complete questionnaire and the dataset supporting the findings of this study are available in Mendeley Data.^18^

### Questionnaire structure

The final version of the questionnaire, developed in Google Forms, consisted of three sections:

1. Demographic and professional information: including region of practice, graduation year, and whether the respondent had completed postgraduate training.
2. CAD/CAM users: questions focused on frequency of use, training received, and clinical experience with technology.
3. Nonusers: questions addressing reasons for nonadoption and perceived limitations.

### Statistical analysis

Survey data were exported into an electronic database (Excel), and analyses were performed using Jamovi software (version 2.6.26). Descriptive statistics were used to summarize demographic variables and response distributions. The Chi-square test was applied to assess associations between categorical variables.

Two binary logistic regression models were conducted to examine factors associated with CAD/CAM adoption (yes/no). In Model 1, all demographic variables: years since graduation, region of practice, postgraduate education, and type of practice were entered as independent variables. In Model 2, type of practice was entered as the main predictor while controlling for the remaining covariates. All variables were entered simultaneously using the Enter method; no forward, backward, or stepwise variable selection procedures were applied.

Model performance and goodness-of-fit were evaluated using the overall model *χ*² statistic, Nagelkerke *R*², Akaike Information Criterion, and Bayesian Information Criterion. Both models converged without errors and demonstrated statistically significant global fit.

Results are reported as odds ratios (OR) with 95% confidence intervals (CI). A *p* value <.05 was considered statistically significant.

## Results

A total of 374 dentists completed the survey. Most respondents had 0 to 10 years since graduation (45.5%) and were concentrated in the National District (37.4%). More than half reported holding a postgraduate degree (56.7%). The majority worked in private practice (53.7%), while 16.8% worked in the public sector. Overall, 39.3% reported using CAD/CAM technology (Table 1).

**Table 1:** Sociodemographic and practice-related characteristics of participating dentists (*n* = 374).

Variable/category	Absolute (*n*)	Relative (%)
**Years of experience**		
0-10 y	170	45.5
11-20 y	107	28.6
21-30 y	60	16.0
More than 30 y	37	9.9
**Region**		
Distrito Nacional	140	37.4
Northern Region	95	25.4
Eastern Region	88	23.5
Southern Region	51	13.6
**Specialty (Master/PhD)**		
Yes	212	56.7
No	162	43.3
**Specialty area**		
Prosthodontics	39	18.5
Orthodontics	37	17.5
Periodontics	37	17.5
Oral surgery	34	16.1
Endodontics	23	10.9
Operative dentistry	16	7.6
Paediatric dentistry	16	7.6
Other	9	4.3
**Type of practice**		
Private (fee-paying patients)	201	53.7
Public practice	63	16.8
Private (insured patients)	60	16.0
University professor	37	9.9
Other	13	3.5
**CAD/CAM use**		
Yes	147	39.3
No	227	60.7

Values are presented as absolute frequencies (*n*) and percentages (%), calculated in relation to the total sample (*n* = 374).

Chi-square analyses showed a significant association between adoption and both practice type and postgraduate education (*p* < .001). In contrast, no associations were found with years since graduation (*P* = .740) or with the region of practice (*p* = .464) (Table 2). Specialists in prosthodontics, orthodontics, and periodontics reported the highest prevalence of CAD/CAM use, while endodontics and paediatric dentistry reported lower adoption rates.

**Table 2:** Sociodemographic and practice-related characteristics according to CAD/CAM use (*n* = 374).

Variable/category	CAD/CAM nonusers *n* (%)	CAD/CAM users *n* (%)	Total *n* (%)	*χ*² (df)		*P* value
**Years of experience**				0.84 (3)		.840
0-10 y	103 (45.4)	67 (45.6)	170 (45.5)			
11-20 y	62 (27.3)	45 (30.6)	107 (28.6)			
21-30 y	39 (17.2)	21 (14.3)	60 (16.0)			
More than 30 y	23 (10.1)	14 (9.5)	37 (9.9)			
**Region**				2.56 (3)		.464
Distrito Nacional	78 (34.4)	62 (42.2)	140 (37.4)			
Northern Region	61 (26.9)	34 (23.1)	95 (25.4)			
Eastern Region	57 (25.1)	31 (21.1)	88 (23.5)			
Southern Region	31 (13.7)	20 (13.6)	51 (13.6)			
**Specialty (Master/PhD)**				23.47 (1)		**<.001**
Yes	106 (46.7)	106 (72.1)	212 (56.7)			
No	121 (53.3)	41 (27.9)	162 (43.3)			
**Specialty area**				29.12 (7)		**<.001**
Prosthodontics	11 (10.5)	28 (26.4)	39 (18.5)			
Orthodontics	12 (11.4)	25 (23.6)	37 (17.5)			
Periodontics	17 (16.2)	20 (18.9)	37 (17.5)			
Oral surgery	22 (21.0)	12 (11.3)	34 (16.1)			
Endodontics	18 (17.1)	5 (4.7)	23 (10.9)			
Operative dentistry	7 (6.7)	9 (8.5)	16 (7.6)			
Paediatric dentistry	13 (12.4)	3 (2.8)	16 (7.6)			
Other	5 (4.8)	4 (3.8)	9 (4.3)			
**Type of practice**				29.46 (4)		**<.001**
Private (fee-paying patients)	105 (46.3)	96 (65.3)	201 (53.7)			
Public practice	54 (23.8)	9 (6.1)	63 (16.8)			
Private (insured patients)	31 (13.7)	29 (19.7)	60 (16.0)			
University professor	29 (12.8)	8 (5.4)	37 (9.9)			
Other	8 (3.5)	5 (3.4)	13 (3.5)			

Values are presented as absolute frequencies (*n*) and column percentages (%). Percentages were calculated within CAD/CAM user groups. Pearson’s chi-square tests were applied, except for specialty area, where Fisher’s exact test was used due to low expected counts. Significant results (*P* < .05) are highlighted in bold.

Multivariable logistic regression confirmed that prosthodontics and orthodontics were the strongest predictors of CAD/CAM adoption (OR = 9.16 and OR = 7.50, respectively), followed by operative dentistry and periodontics. Endodontics was negatively associated (OR = 0.28). Regarding practice setting, dentists in public institutions (OR = 0.18) and those in academic roles (OR = 0.28) were significantly less likely to use CAD/CAM compared with private practitioners (Table 3).

**Table 3:** Multivariable logistic regression for specialty and type of practice associated with CAD/CAM use.

Predictor	OR	95% CI for OR	*P* value
**Specialty area**			
General dentist [reference]	___	___	___
Prosthodontics	9.16	[2.73-30.78]	**<.001**
Orthodontics	7.50	[2.24-25.06]	**.001**
Operative dentistry	4.63	[1.14-18.75]	**.032**
Periodontics	4.24	[1.30-13.83]	**.017**
Oral surgery	1.96	[0.58-6.62]	.276
Endodontics	0.28	[0.10-0.75]	**.011**
Paediatric dentistry	0.83	[0.17-4.11]	.82
Other	2.88	[0.56-14.94]	.208
**Type of practice**	**___**	***___***	***___***
Private (fee-paying patients) [reference]	0.91	[0.69-1.21]	.53
Public practice	0.18	[0.08-0.34]	**<.001**
Private (health insurance patients)	0.94	[0.56-1.55]	.80
University professor	0.28	[0.13-0.60]	**.001**
Other	0.63	[0.20-1.91]	.41

Odds ratios (OR) are adjusted for all other variables in the model. The reference category for specialty is General dentist. The reference category for type of practice is Private practice (fee-paying patients). Significant results (*P* < .05) are highlighted in bold.

CI, confidence interval.

**Fig. 1:**
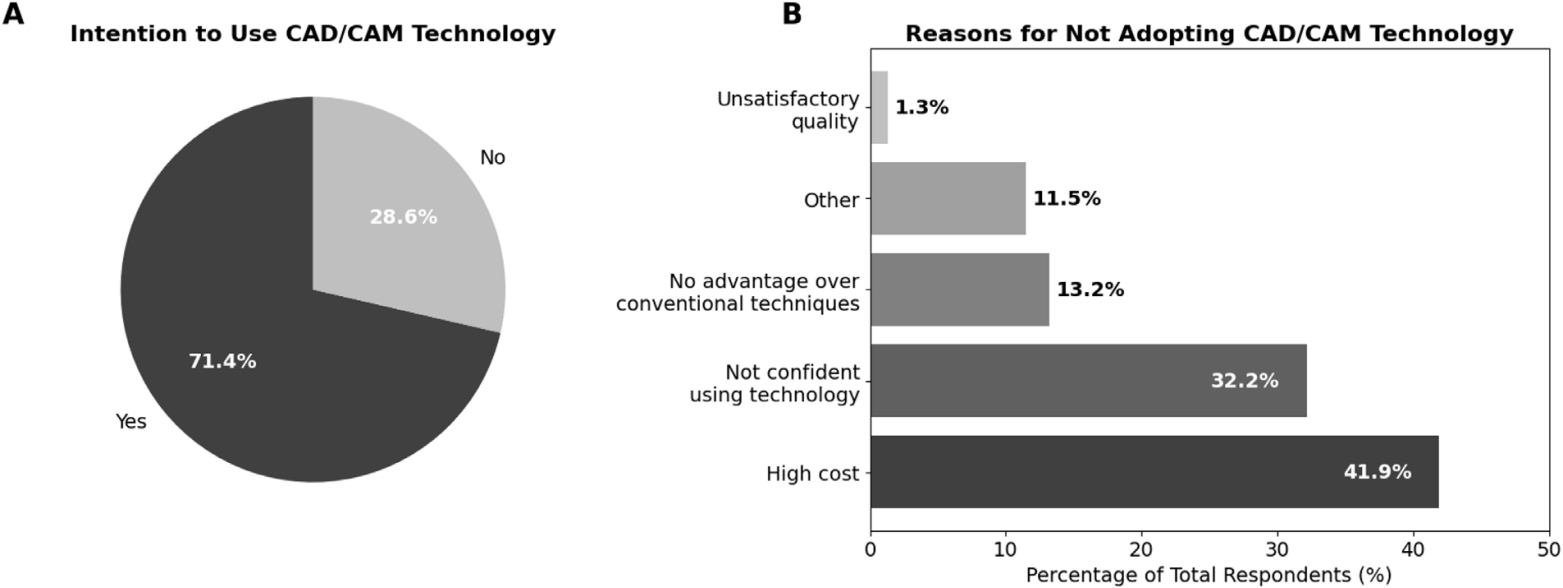
(A and B) Intention to adopt CAD/CAM technology and perceived barriers among nonusers (*n* = 227). Part (A) shows the proportion of nonusers who reported intention to adopt CAD/CAM technology in the future (‘Yes’ vs ‘No’). Part (B) presents the distribution of perceived barriers to adoption, including high cost, limited confidence using the technology, no perceived advantage over conventional techniques, lack of relevance to their clinical scope, and concerns regarding quality. Bars represent percentages of the total number of nonusers, and the corresponding absolute frequencies are displayed above each category.

Among nonusers, high cost (41.9%) and limited confidence in using the technology (32.2%) were the most frequently cited barriers, while 71.4% expressed intention to adopt CAD/CAM into their future. These patterns varied by practice type and years of experience. Figure 1A illustrates adoption intentions among nonusers, while Figure 1B summarizes the distribution of reported barriers. Qualitative responses reinforced these findings, citing lack of access to equipment, eg, *‘I don’t have my own practice’, ‘The practice where I work does not offer the service’*, limited training, eg, *‘I’m not fully familiar with it’, ‘I don’t know much about the scanning technology’*, or perceived irrelevance to their scope of practice, eg, *‘It doesn’t apply to my area of specialization’, ‘It’s not essential for the work I do”*. Some expressed uncertainty about the quality of CAD/CAM restorations, while others indicated not having the opportunity to use the technology.

**Figure 2:**
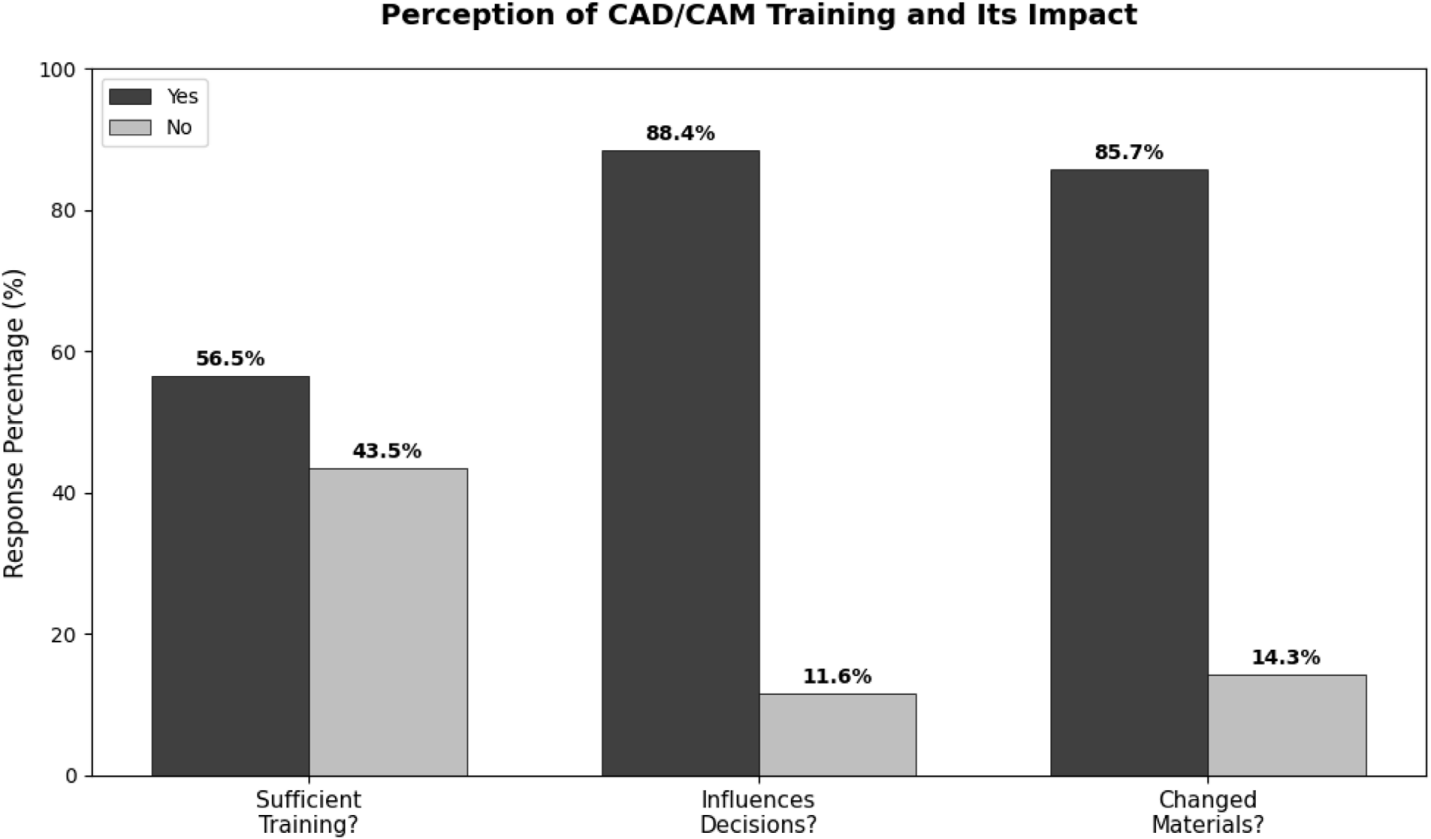
Perception of CAD/CAM training sufficiency and its association with changes in clinical practice among current users (*n* = 147). The figure summarizes responses to three yes/no items evaluating: (1) whether users considered their CAD/CAM training sufficient, (2) whether CAD/CAM use influenced their clinical decision-making, and (3) whether it led to changes in material selection. Bars represent the percentage of respondents selecting each option (‘Yes’ or ‘No’), with absolute frequencies displayed above each bar.

Among CAD/CAM users, most had adopted the technology within the past 5 years. Just over half considered their training was sufficient, and those who considered their training adequate were significantly more likely to report changes in clinical decision-making (*p* = .004) and material selection (*p* = .005). Figure 2 presents users’ perceptions of training sufficiency and its association with changes in practice behaviour.

Motivations for CAD/CAM adoption included improving quality (17.6%), staying technologically up to date (15.1%), and enhancing patient experience (15.3%). Achievements aligned with these goals, including remaining technologically current (22.7%) and improved productivity (20.6%). A summary of these distributions is presented in Supplementary Table S1.

Regarding digital workflow, the most frequently used components were intraoral scanners (27.8%) and lab-based scanning of impressions or models (21.8%). The most commonly used CAD/CAM materials were lithium disilicate (13.8%), resin (12.5%), and monolithic zirconia (10.7%). The main clinical indications included crowns (15.2%), bridges (14.2%), implant restorations (10.4%), and smile design (10.3%). Additional details on CAD/CAM workflow components, materials used, and clinical procedures performed are summarized in Supplementary Table S2.

Finally, additional binary logistic regression models were conducted to confirm the association between postgraduate education and CAD/CAM use (OR = 2.95; 95% CI [1.90-4.64]; *P* < .001) and between type of practice and CAD/CAM use (OR = 0.25; 95% CI [0.06-1.13]; *P* = .061).

## Discussion

This first national survey on CAD/CAM adoption in the Dominican Republic demonstrates a moderate adoption rate, with specialty and practice setting emerging as the strongest predictors of use. Prosthodontics and orthodontics showed the highest likelihood of adoption, consistent with the procedural demands and restorative workflows characteristic of these specialties.^1^^,^^2^^,^^8^ Operative dentistry also appeared as a significant predictor, likely reflecting the expansion of digital workflows into indirect adhesive and esthetic procedures, whereas endodontics remained negatively associated, an expected pattern given the limited relevance of CAD/CAM to endodontic treatment pathways. The participation of general practitioners further suggests that digital workflows are expanding beyond traditional specialist-driven contexts, indicating an early diffusion into broader clinical practice.^11^^,^^15^

International comparisons reinforce these interpretations. Studies from the UK, Egypt, and Saudi Arabia consistently show higher adoption among prosthodontists and private practice settings.^9^^,^^11^^,^^13^ Although early-career dentists expressed high interest in CAD/CAM, a trend also observed globally, cost and infrastructural limitations remain major constraints, limiting translation of interest into practice.^10^^,^^14^^,^^16^ These trends align with broader analyses showing that digitalization is reshaping educational priorities and competency requirements internationally.^7^

Financial limitations were the most frequently cited barrier, mirroring evidence from other settings where high initial investment and maintenance demands, and limited institutional support restrict digital transitions.^9^^,^^19^ In contexts where equipment is individually financed, economic feasibility becomes particularly more restrictive. This reinforces the importance of developing financing strategies, subsidies, or shared-access models to facilitate adoption, particularly among early-career clinicians and practices serving lower-income populations.^15^^,^^16^

Training emerged as a second major barrier. Only 56.5% of Dominican users felt adequately trained, and training sufficiency was associated with differences in decision-making and material selection. Similar concerns have been documented internationally, where many clinicians rely on self-directed learning or short courses rather than structured educational programs.^9^^,^^19^ Strengthening undergraduate, postgraduate, and continuing education in digital dentistry is therefore essential to ensure safe and effective implementation.^14^^,^^20^ Recent European analyses have likewise emphasized the need for more robust digital competencies in formal curricula.^7^

Practice setting exerted a strong influence on adoption. Digital systems remain uncommon in Dominican public dental services, rendering integration more feasible in private practice. Comparable disparities have been documented in Saudi Arabia, the UK, China, and India, where private clinics demonstrate greater readiness to adopt new technologies.^9^^,^^10^^,^^11^^,^^21^ Policy-level initiatives and investment in public sector digital infrastructure could reduce these structural inequities and support more balanced national integration.

The predominance of CAD/CAM use for crowns, bridges, and implant-supported prostheses reflects established indications supported by a strong evidence base.^4^^,^^13^^,^^15^^,^^20^ Patient experience, including reduced discomfort from impression, fewer clinical visits, and the possibility of same-day restorations, likely contribute to positive perceptions and clinical acceptance. As hybrid ceramics and polymer-based CAD/CAM materials continue evolving, broader adoption can be expected, particularly if training and cost barriers are addressed.^2^^,^^20^

This study has several limitations. The convenience sampling strategy and online distribution may have introduced selection bias favouring digitally engaged respondents, and self-reported data inherently subject to recall and social desirability bias. The absence of differentiation between chairside and laboratory-based CAD/CAM modalities limits the workflow comparisons. Additionally, aspects such as technical support, maintenance requirements, and case-specific limitations were not examined, although international literature consistently identifies these as relevant factors affecting long-term use.^12^^,^^15^ As a cross-sectional design, causal inferences cannot be established.

Despite these limitations, this study provides novel data from the underrepresented Caribbean region. These insights emphasize the need to improve training, expand equitable access across practice settings, and develop policy strategies that support the effective and sustainable integration of digital workflows into dental practice.

## Conclusion

This study provides the first national assessment of CAD/CAM use among dentists in the Dominican Republic, revealing significant associations with specialty and practice type. Economic constraints and limited training emerged as the main barriers to adoption, yet most nonusers expressed interest in future implementation. These findings underscore the need for targeted strategies to improve access to training, reduce financial barriers, and promote equitable integration of digital technologies into dental practice.

## Ethics statement

This study was approved by the Comité de Ética de Investigación de la Universidad Iberoamericana (CEI-UNIBE), approval code: CEI2024-0589. This study was conducted in accordance with the Declaration of Helsinki. Participation was voluntary and anonymous, and digital informed consent was obtained prior to survey completion.

## Funding

This research did not receive any specific grant from funding agencies in the public, commercial, or not-for-profit sectors.

## Author contributions

Patricia Grau Grullón contributed to conceptualization, methodology, investigation, project administration, resources, supervision, visualization, writing – original draft, and writing – review and editing. Rodrigo Varella Carvalho was responsible for data curation, formal analysis, and writing – review and editing. Gabriela Velázquez Pérez contributed to validation, data curation, and investigation. Leyani Perez and Roxana Crespo contributed to investigation. Giana Lima participated in validation and writing – review and editing.

## Declaration of generative AI and AI-assisted technologies in the writing process

During the preparation of this work, the authors used ChatGPT (OpenAI, version Sept 2025) to improve language clarity. After use, the content was reviewed and edited, and the authors take full responsibility for the content of the publication.

## Conflict of interest

The authors declare that they have no known competing financial interests or personal relationships that could have appeared to influence the work reported in this article.

## Appendix Supplementary materials

Image, application 1

## Footnotes

Supplementary material associated with this article can be found in the online version at doi:10.1016/j.identj.2026.109411.
