# Supplementary material for: Adoption and Barriers to CAD/CAM Technology Among Dentists in the Dominican Republic: A Cross-Sectional Study
Source: Int Dent J. 2026 Apr 2;76(3):109411. doi: 10.1016/j.identj.2026.109411 (PMC13085077; doi:10.1016/j.identj.2026.109411)
Supplement: Supplementary file 1 [file mmc1.docx]

**Supplementary Table S1.** Motivations for adopting CAD/CAM technology and reported achievements among current users in the Dominican Republic.

| **Reason** | **Frequency** | **Valid Percent** |
| --- | --- | --- |
| Improve quality | 94 | 17.6 |
| To improve the patient experience in the consultation | 82 | 15.3 |
| To be technologically up to date | 81 | 15.1 |
| Increase productivity | 72 | 13.5 |
| Use new dental materials that can only be manufactured using CAD/CAM | 60 | 11.2 |
| To improve communication with the dental laboratory | 57 | 10.7 |
| To reduce manufacturing costs | 51 | 9.5 |
| For better promotion and presence on social networks | 38 | 7.1 |
| Missing | 131 | - |
| **Total** | **666** | **100.0** |

| **Achievement** | **Frequency** | **Valid Percent** |
| --- | --- | --- |
| Be technologically up to date | 135 | 22.7 |
| Improve productivity | 123 | 20.6 |
| Marketing to attract patients | 99 | 16.6 |
| Improve communication with the dental laboratory | 96 | 16.1 |
| Improve quality | 91 | 15.3 |
| Reduce manufacturing costs | 49 | 8.2 |
| Provide updates and more comfort for patients | 2 | 0.3 |
| Save time | 1 | 0.2 |
| Missing | 70 | - |
| **Total** | **666** | **100.0** |

*Multiple responses were allowed; therefore, the total number of responses (n = 666) exceeds the number of CAD/CAM users (n = 147). Percentages represent valid percent values, calculated over the total number of selections for each item (summing to 100%).*

**Supplementary Table S2.** Digital workflow components, materials used, and clinical procedures performed with CAD/CAM systems among dentists in the Dominican Republic.

| **Digital workflow aspect** | **Frequency** | **Valid Percent** |
| --- | --- | --- |
| Intraoral scanner | 107 | 27.8 |
| Scanning impressions and models in the laboratory | 84 | 21.8 |
| Computer-aided design (CAD by specialized dental laboratory) | 83 | 21.6 |
| Computer-aided manufacturing (CAM by specialized dental laboratory) | 64 | 16.6 |
| In-office CAD/CAM devices such as CEREC | 47 | 12.2 |
| Missing | 281 | - |
| **Total** | **666** | **100.0** |

| **Material** | **Frequency** | **Valid Percent** |
| --- | --- | --- |
| Lithium disilicate | 92 | 13.8 |
| Resin | 83 | 12.5 |
| Monolithic zirconium | 71 | 10.7 |
| PMMA Milled Metals | 61 | 9.2 |
| Layered zirconium | 59 | 8.9 |
| Zirconium-reinforced glass ceramics | 58 | 8.7 |
| Leucite/feldspar glass ceramics | 49 | 7.4 |
| Nanoceramic | 47 | 7.1 |
| Zirconium oxide | 47 | 7.1 |
| Plastic | 41 | 6.2 |
| Millable waxes | 31 | 4.7 |
| Metals | 27 | 4.1 |
| Missing | 0 | - |
| **Total** | **666** | **100.0** |
| **Procedure** | **Frequency** | **Valid Percent** |
| Dental crowns | 101 | 15.2 |
| Dental bridges | 94 | 14.2 |
| Dental casts | 71 | 10.7 |
| Inlays/onlays | 69 | 10.4 |
| Restorations on implants | 69 | 10.4 |
| Smile design | 68 | 10.3 |
| Invisalign | 57 | 8.6 |
| Complete and partial dentures | 49 | 7.4 |
| Guided surgeries | 46 | 6.9 |
| Orthodontics | 38 | 5.7 |
| Other | 1 | 0.2 |
| Missing | 3 | - |
| **Total** | **666** | **100.0** |

*Multiple responses were allowed; therefore, the total number of responses (n = 666) exceeds the number of CAD/CAM users (n = 147). Percentages represent valid percent values, calculated over the total number of selections for each item (summing to 100%).*
